# The N-end rule and retroviral infection: no effect on integrase

**DOI:** 10.1186/1743-422X-10-233

**Published:** 2013-07-13

**Authors:** Guney Boso, Takafumi Tasaki, Yong Tae Kwon, Nikunj V Somia

**Affiliations:** 1 Developmental Biology and Genetics Graduate Program, Molecular, Cellular, University of Minnesota, Minneapolis, MN, USA; 2 Medical Research Institute, Kanazawa Medical University, Ishikawa, Japan; 3 Center for Pharmacogenetics and Department of Pharmaceutical Sciences, University of Pittsburgh, Pittsburgh, USA; 4 Department of Biomedical Sciences, College of Medicine, Seoul National University, Seoul 110-799, Korea; 5 Department of Genetics, Cell Biology and Development, University of Minnesota, 6-160 Jackson Hall, 321, Church St., SE, Minneapolis, MN 55455, USA

**Keywords:** N-end rule, HIV-1, MLV, Integrase

## Abstract

**Background:**

Integration of double stranded viral DNA is a key step in the retroviral life cycle. Virally encoded enzyme, integrase, plays a central role in this reaction. Mature forms of integrase of several retroviruses (i.e. HIV-1 and MLV) bear conserved destabilizing N-terminal residues of the N-end rule pathway - a ubiquitin dependent proteolytic system in which the N-terminal residue of a protein determines its half life. Substrates of the N-end rule pathway are recognized by E3 ubiquitin ligases called N-recognins. We have previously shown that the inactivation of three of these N-recognins, namely UBR1, UBR2 and UBR4 in mouse embryonic fibroblasts (MEFs) leads to increased stability of ectopically expressed HIV-1 integrase. These findings have prompted us to investigate the involvement of the N-end rule pathway in the HIV-1 life cycle.

**Results:**

The infectivity of HIV-1 but not MLV was decreased in N-recognin deficient cells in which three N-recognins (UBR1, UBR2 and UBR4) were depleted. HIV-1 integrase mutants of N-terminal amino acids (coding for stabilizing or destabilizing residues) were severely impaired in their infectivity in both human and mouse cells. Quantitative PCR analysis revealed that this inhibition was mainly caused by a defect in reverse transcription. The decreased infectivity was independent of the N-end rule since cells deficient in N-recognins were equally refractory to infection by the integrase mutants. MLV integrase mutants showed no difference in their infectivity or intravirion processing of integrase.

**Conclusions:**

The N-end rule pathway impacts the early phase of the HIV-1 life cycle; however this effect is not the result of the direct action of the N-end rule pathway on the viral integrase. The N-terminal amino acid residue of integrase is highly conserved and cannot be altered without causing a substantial decrease in viral infectivity.

## Background

Retroviruses are obligate intracellular parasites that must exploit host cell pathways during their life cycle. These activities range from using motor proteins [1], nuclear import [2] and export pathways, to transcription [3] and processing [4] of the viral genome. Studying the role of these cellular pathways in retroviral replication can lead to a deeper understanding of the viral life cycle. This has been highlighted recently with the development of high throughput screening techniques which have implicated numerous proteins that are either required or act to restrict retroviral infection [5-8]. One of the processes repeatedly implicated in several genome wide screens is the ubiquitin proteasome pathway that mediates signal dependent degradation of proteins. Different proteins of this pathway have been found in all four genome wide RNAi screens conducted looking for host factors used by HIV-1 [5-8]. Although over the years, a variety of studies have implicated the ubiquitin proteasome system in the viral life cycle, research on the role of this essential pathway in retroviral infection has mostly been focused on targeting of cellular restriction factors to the proteasome by viral accessory proteins [9-11]. Results of the genomewide studies on host factors suggest that retroviruses may be using the ubiquitin proteasome pathway in the early phase of the viral life cycle.

One of the degradation signals that target cellular proteins to the proteasome is the N-terminal amino acid residue of the protein. First described by Alex Varshavsky and colleagues in 1986, this N-end rule is an ubiquitin dependent proteolytic system in which the identity of the N-terminal amino acid residue of a protein determines its half life in the cell [12]. Even though it was originally discovered while expressing a bacterial protein in yeast cells the N-end rule pathway was subsequently found to be present in all organisms examined including mammals [13], plants [14], and bacteria [15]. Hence it is an evolutionarily ancient mechanism for protein degradation. In mammalian cells, amino acids are classified as stabilizing (Met, Ala, Val, Gly, Pro, Ser, Thr) or destabilizing (Glu, Gln, Cys, Asp, Asn, Arg, Lys, His, Leu, Ile, Phe, Trp, Tyr) based on their ability to act as a degradation signal for the N-end rule pathway. Although the N-end rule classifies most amino acids as destabilizing for the mammalian cells, it has been difficult to identify the cellular substrates of this pathway. However with the discovery of several signaling components of the N-end rule pathway our understanding of this process has increased in the last decade.

Specificity of the ubiquitin proteasome system is governed by E3 ubiquitin ligases that recognize a degradation signal on the target protein and catalyze the addition of ubiquitin onto the protein. For the N-end rule pathway, these ligases are called N-recognins, and the signal for degradation is termed an N-degron [16]. The first N-recognin was discovered in *S. cerevisiae* and termed Ubr1 [16]. While Ubr1 is the sole N-recognin in yeast cells, subsequent studies have identified two homologs of Ubr1 in mammalian genomes, UBR1 and UBR2 [17]. These proteins have been shown to have highly similar sequences and overlapping functions [17,18]. Biochemical studies identified two more E3 ligases that can bind to destabilizing N-terminal residues, termed UBR4 and UBR5 [18]. These proteins contain a common zinc finger like domain termed a UBR box [18]. Mammalian genomes contain three additional genes that code for the UBR box domain, UBR3, UBR6 and UBR7 [18]. However these proteins have not been shown to bind any of the destabilizing residues and therefore their role in the N-end rule pathway, if any, is unknown.

While recent studies on N-recognins have substantially increased our understanding of the N-end rule pathway, identification of the cellular substrates of N-end rule has been challenging. Since almost all mammalian proteins are synthesized with an N-terminal methionine, a stabilizing residue, an N-degron can only be created through post translational modifications, such as the removal of the N-terminal methionine [19] or endoproteolytic cleavage. Proteolytic cleavage of certain viral proteins can generate potential substrates for the N-end rule pathway. One viral protein that has been studied with respect to the N-end rule is the integrase of HIV-1. Retroviral integrase is synthesized as a part of a Gag Pol polyprotein (Pol in spumaretroviruses). Along with other viral proteins, Gag Pol is packaged into viral particle during assembly and, upon viral budding, the mature form of integrase is generated as a result of a series of proteolytic cleavage events mediated by the viral protease. We have previously shown that concomitant impairment of three of the N-recognins that bind to destabilizing residues, UBR1, UBR2 and UBR4 in mouse embryonic fibroblasts (MEFs) leads to increased stability of ectopically expressed HIV-1 integrase bearing a destabilizing residue [18]. These results and others [20,21] raised the possibility that HIV-1 (and possibly other retroviruses) may utilize the N-end rule pathway to control the stability of integrase during viral infection.

In this study we investigated the potential role of the N-end rule pathway during the early phase of the life cycle of two different retroviruses; HIV-1 and MLV. Here we show that HIV-1 but not MLV infectivity is decreased in cells where the N-end rule pathway was impaired. We also show that the N-terminal amino acid residue of HIV-1 integrase which has previously been suggested to be a target for degradation by the N-end rule is not targeted by the N-end rule pathway during infection.

## Results

### Proteolytic cleavage of Gag/Pol generates destabilizing N-end rule residues

**Figure 1:**
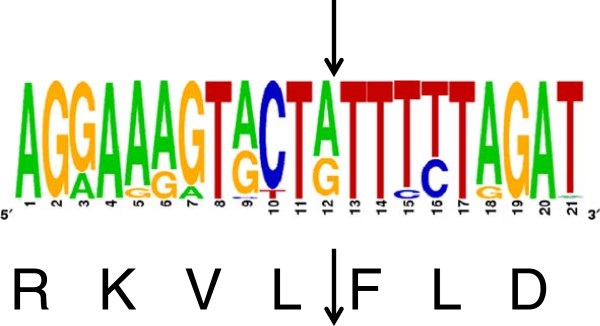
**N terminal residue of HIV integrase is highly conserved.** Analysis of sequence of the protease cleavage site between RT and integrase of 1850 isolates of HIV-1 and SIVcpz present in Los Alamos HIV Database (http://www.hiv.lanl.gov) using the web alignment tool. Corresponding amino acid for the conserved sequence is shown at the bottom. The sequence logo at the top was generated using WebLogo (http://weblogo.berkeley.edu/). Arrows indicate the protease cleavage site.

We first examined whether the cleavage of the Gag Pol polyprotein by HIV-1 protease would generate mature proteins that expose destabilizing N-terminal residues of the N-end rule pathway. Each HIV-1 protease cleavage recognition site in the polyprotein between the subunits differs in its amino acid sequence. Hence the nature of the sequence and its accessibility is thought to determine the specificity for cleavage [22]. Furthermore variation among groups or subtypes of HIV may result in sequence variation of the cleavage site. Therefore, we reasoned that if HIV-1 utilizes the N-end rule in its lifecycle, the N-terminal amino acids of mature protein should be conserved as N-degrons across different types, groups and subgroups of HIV-1. The conservation of N-terminal destabilizing residues across other lentiviruses would also suggest a selection pressure to maintain their interaction with N-end rule machinery. A previous study has compared HIV-1 type 1 protease cleavage sites and identified the most common recent common ancestor (MRCA) of protease cleavage sites in HIV-1 for groups M, B and C [23]. Of note, 7 proteins are expected to bear N-terminal destabilizing residues. We have further analyzed the conservation of the N-terminal residues of these 7 proteins by comparing the sequences of 1850 HIV-1 isolates from the Los Alamos HIV Sequence Database (http://www.hiv.lanl.gov/). Of the sequences analyzed we have found that 4 proteins (p1, Trans frame octapeptide (TFP), RNaseH and Integrase) have N-terminal amino acid sequences that are conserved relative to the MRCA. The data is summarized in Table 1 with respect to the N-terminal amino acid generated and its predicted behavior according to the N-end rule. Result for the cleavage site between Reverse Transcriptase and Integrase is shown in Figure 1. In this study we decided to focus on the integrase molecule because it was implicated as a target for the N-end rule [18,20,21] and since it is the focus of small molecule development to combat HIV-1.

**Table 1:** HIV-1 proteins have N-terminal destabilizing residues

**HIV-1 cleavage site and conservation**	**Amino acid at the N terminus**	**N-end rule designation**
MA/CA Conserved	Proline	Stabilizing
CA/p2 Conserved	Alanine	Stabilizing
P2/NC Variable	Methionine	Stabilizing
**NC/p1 Conserved**	**Phenylalanine**	**Primary Destabilizing**
p1/p6(gag) Variable	Leucine	Primary Destabilizing
**NC/TFP Conserved**	**Phenylalanine**	**Primary Destabilizing**
TFP/p6(pol) Variable	Leucine	Primary Destabilizing
p6(pol)/PR Variable	Proline	Stabilizing
PR/RT Conserved	Proline	Stabilizing
**RT/RH(p15) Conserved**	**Tyrosine**	**Primary Destabilizing**
**RH(p15)/INT Conserved**	**Phenylalanine**	**Primary Destabilizing**
Nef Variable	Leucine	Primary Destabilizing

Comparison of 84 protease cleavage site sequences (27 C-type, 30 B-type and 27 M-type) [23]. The position and the conservation relatives to the most common recent common ancestor (MRCA) are shown. The proteins that are conserved for a destabilizing residue as confirmed by an analysis of 1850 isolates of HIV-1 and SIVcpz present in Los Alamos HIV Database (http://www.hiv.lanl.gov) are highlighted in bold.

### The N-end rule can impact HIV-1 infection

We next tested if the N-end rule can impact HIV-1 infection. To this end we utilized single cycle HIV-1 reporter vectors and cell lines mutant in N-recognins. We have previously characterized redundant functions of the UBR proteins and hence generated cell lines singly or multiply mutant for UBR proteins [18]. These are MEFs from UBR1−/−, UBR2−/− and UBR1−/− UBR2−/− strains of mice. We have also previously generated MEF lines that are deficient in all three UBR box proteins (UBR1, UBR2 and UBR4) that have been shown to bind type 2 destabilizing residues by transducing UBR1−/− UBR2−/− cell lines with 3 different shRNAs against UBR4 mRNA (to account for any off target effects) and a shRNA targeting luciferase mRNA as a control. UBR4 knockdown was verified by immunoblotting [18]. Wild-type MEFs transduced with shRNA to UBR4 mRNA were used to investigate the effect of UBR4 knockdown alone.

**Figure 2:**
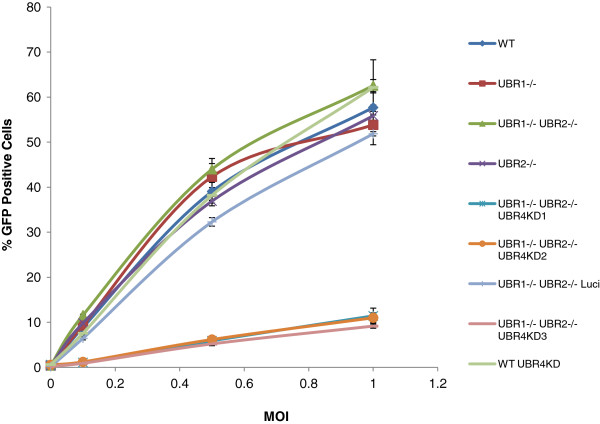
**HIV-1 infectivity is decreased when all three N-recognins are depleted.** WT and N-recognin deficient MEFs were infected with VSVG pseudotyped HIV-1-EGFP at an increasing MOI of 0.1, 0.5 and 1. The percent of GFP positive cells were determined by fluorescence cytometry 3 days post infection.

We infected N-recognin deficient MEFs with a HIV-1 EGFP vector pseudotyped with VSVG. Infectivity was reported as EGFP expression and was scored by flow cytometry 72hrs after infection. Figure 2 illustrates that the infectivity of HIV-1 was unchanged over a range of MOIs (Multiplicity of Infection) comparing wild type (WT), MEFs for UBR1 −/−, UBR2 −/−, UBR4 KD, and the double deletion MEF UBR1 −/− UBR2 −/−. We observed a substantial decrease (5–6 fold) only in cells that are depleted in UBR1, UBR2 and UBR4. We confirmed that this decrease in infectivity is not due to a defect in the reporter or the expression of the reporter by using a different reporter (DS-Red) and a different promoter (CMV) (Additional file 1: Figure S1).

### HIV-1 infection is impaired at reverse transcription or uncoating in N-recognin deficient cells

**Figure 3:**
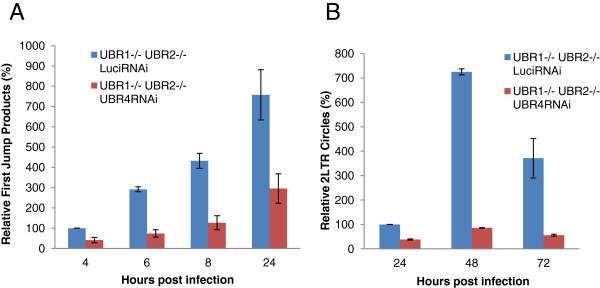
**Kinetics and nuclear localization of proviral DNA in N-recognin deficient cells.** UBR1−/− UBR2−/− Luci RNAi (control) and UBR1−/− UBR2−/−UBR4 RNAi cells were infected with VSVG pseudotyped HIV-1-EGFP at an MOI of 1. Viral cDNA products were quantified by qPCR at indicated times. **(A)** First jump products. The data are expressed as relative to the control 4 hr time point. **(B)** 2LTR circles. The data are expressed as relative to the control 24 hr time point. The data for the UBR1−/− UBR2−/−UBR4RNAi are the average of UBR1−/− UBR2−/−UBR4KD1 and UBR1−/− UBR2−/−UBR4KD2 cells.

These results indicate that N-end rule pathway can impact the early phase of the HIV-1 life cycle. However decrease in infectivity by itself is not enough to determine whether the N-end rule acts on HIV-1 proteins or a cellular protein that is involved in infection. To better pinpoint the stage that is impaired we determined which step in the viral life cycle is affected in the N-recognin deficient MEFs. Following infection we isolated total DNA from MEFs at different time points and tracked the relative accumulation of early reverse transcription products and 2LTR circles by qPCR. As shown in Figure 2 the infection in WT cells compared to UBR1−/− UBR2−/− cells are similar. Therefore we chose to compare UBR1−/− UBR2−/− Luci RNAi to UBR1−/− UBR2−/− UBR4 RNAi cells. As shown in Figure 3A, UBR1−/− UBR2−/− UBR4 RNAi cells showed around 3–4 fold decrease in the accumulation of first jump products. Moreover the rate of increase in the accumulation of first jump products were found to be lower in the triply deficient cells especially in the early time points (compare 4 hour and 6 hour time points) indicating a defect in reverse transcription. However the first point we analyzed also showed around 2 fold difference in the amount of first jump products between the control and the triply deficient cells, which suggests that another defect before the first jump step of the reverse transcription either at the start of reverse transcription or during uncoating could contribute to this phenomenon. The difference we observed in the accumulation of reverse transcription products was further enhanced when we looked at the relative production of 2LTR circles. As illustrated in Figure 3B, the UBR1−/− UBR2−/− UBR4RNAi cells exhibited up to 7 fold less 2LTR circles compared to the control cells. However, since the 2LTR circles are only a subset of the reverse transcription products that enter the nucleus we do not observe any defect in nuclear import. Furthermore it has been reported that in the presence of mutant non-functional integrase 2LTR circles are increased when compared to infection with wild type integrase [24,25]. Hence we compared the ratio of first jump to 2LTR circles at 24 hrs. The data show that for UBR1−/− UBR2−/− LuciRNAi control cells the ratio is 216.7 ± 34.6, while the ratio in triply mutant cells is 250.3 ± 53.9. This indicates that the integrase function is not impaired in the triply mutant cells. We conclude that the triply deficient cells are impaired in early reverse transcription or uncoating.

### The N-end rule does not impact MLV infection

**Figure 4:**
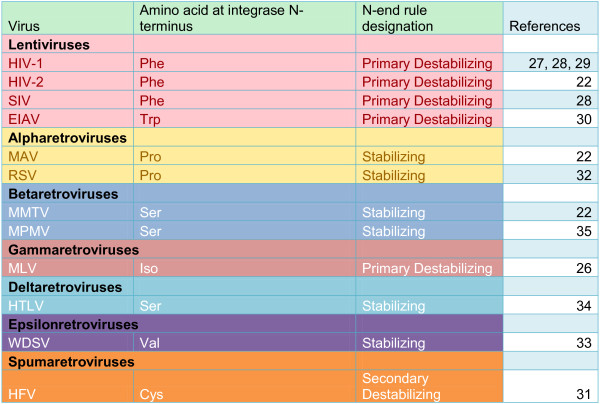
**Only some retroviral integrases bear a N-terminal type-2 destabilizing residue.** A comparison of the identity and nature of the amino acid at the N-terminus of mature integrase for various retroviruses. Representatives of the family of retroviruses were chosen based on sequence characterization of the cleavage site or the integrase protein from the literature [22,26-35].

**Figure 5:**
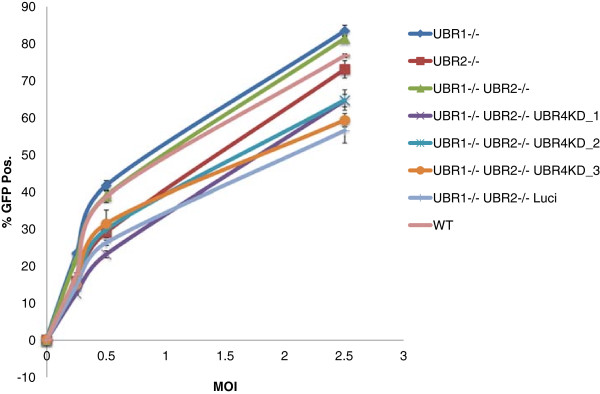
**MLV infectivity is not affected in N-recognin deficient cell.** WT and N-recognin deficient MEFs were infected with VSVG pseudotyped MLV-EGFP at an increasing MOI of 0.25, 0.5 and 2.5. The percent of GFP positive cells were determined by fluorescence cytometry 3 days post infection.

Similar to HIV-1, proteins of other retroviruses also harbor N-terminal residues that make them susceptible to the N-end rule. For example mature MLV integrase also bears a type 2 destabilizing residue isoleucine making it a potential substrate for the N-end rule pathway [26]. A comparison of different retroviral integrase N-terminal residues revealed that all lentiviruses examined, gammaretroviruses and spumaretroviruses harbor destabilizing residues. However, this is not universal to retroviruses, since alpha, beta, delta and epsilon retroviruses harbor a stabilizing residue (Figure 4). To test whether MLV infection is affected by the impairment of the N-end rule pathway, we infected our N-recognin deficient MEFs using a MLV based EGFP vector pseudotyped with VSVG. In contrast to HIV-1, MLV infectivity was only slightly decreased in UBR1−/− UBR2−/− UBR4KD cells compared to the other cells tested (Figure 5). Notably the slight decrease was also present in the control UBR1 −/−, UBR2 −/−, Luciferase RNAi cell line. This leads us to conclude that the impact of the N-end rule on MLV infection is minimal. This also allows us to conclude that the decrease observed for HIV-1 on the triply deficient cells is not due to an entry defect since the MLV and HIV vectors are pseudotyped with VSVG and hence utilize the same mode of entry.

### The N-terminal residue impacts intravirion stability of integrase

**Figure 6:**
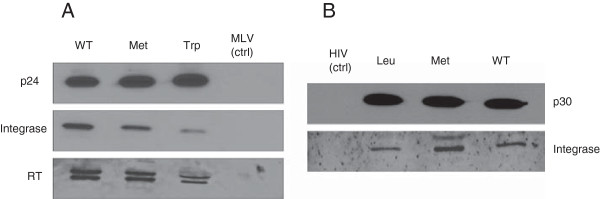
**Intravirion integrase levels are decreased in HIV-1 integrase N-terminal mutants.** HIV-1 or MLV virions were produced in 293T cells using helper plasmids with either WT or engineered mutations for the N-terminus of mature integrase. Virions particles were concentrated and capsid equivalent (p24 for HIV-1 and p30 for MLV) extracts were analyzed by immunoblot analysis. **A)** HIV-1 vector particle extracts probed with p24, integrase or RT antibodies. The MLV (ctrl) particle extract serves as a negative control. **B)** MLV vector particle extracts probed with p30 or MLV integrase antibodies. The HIV (ctrl) extract serves as a negative control.

The observed differential effect of N-recognin deficiency on HIV-1 and MLV prompted us to question the previous suggestions that HIV-1 integrase stability may be controlled by the N-end rule pathway during viral life cycle. To better assess the possible involvement of the N-end rule pathway in controlling integrase stability during retroviral infection, we generated N-terminal integrase point mutants of HIV-1 and MLV with an N-terminal stabilizing or destabilizing residue within the context of the GagPol protein (see Tables 2 and 3). For both viruses we chose methionine as the stabilizing residue since HIV-1 mutants with this residue at the N-terminus have previously been shown to produce virions [21]. Both HIV-1 and MLV mature integrases bear a type 2 destabilizing residue at their N-terminus [26]. Hence, we also substituted the WT destabilizing residues of both viruses with another, structurally similar type 2 destabilizing residue as an additional control. For HIV-1 we replaced phenylalanine at the N-terminus of integrase with another aromatic amino acid, tryptophan (Table 2). For the integrase of MLV, we replaced isoleucine with another branched chain amino acid, leucine (Table 3). In order to evaluate correct packaging and maturation of viral proteins in our integrase mutants, we analyzed viral pellets by immunoblotting. As shown in Figure 6A, HIV-1 integrase mutants successfully produced viral particles as evidenced by the presence of the mature capsid protein (p24) in the concentrated viral pellets. However, the amount of integrase was decreased in these mutants compared to WT virus. Levels of reverse transcriptase subunits found in the viral particles paralleled the decreased levels of integrase. In contrast levels of integrase were not significantly altered for the MLV integrase mutants (Figure 6B).

**Table 2:** N-terminal integrase mutants of HIV-1

**Cleavage site (RH/INT)**	**N-end rule designation**	**Amino acid at P1’**
RKVL/FLD (WT)	Destabilizing	Phenylalanine
RKVL/MLD	Stabilizing	Methionine
RKVL/WLD	Destabilizing	Tryptophan

**Table 3:** N-terminal integrase mutants of MLV

**Cleavage site (RH/INT)**	**N-end rule designation**	**Amino acid at P1’**
STLL/IEN (WT)	Destabilizing	Isoleucine
STLL/MEN	Stabilizing	Methionine
STLL/LEN	Destabilizing	Leucine

### HIV-1 Integrase mutants package equivalent amounts of GagPol

**Figure 7:**
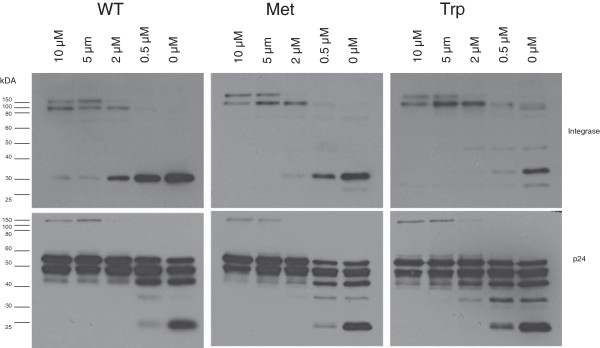
**Intravirion processing of Gag and Gag-Pol polyproteins in HIV-1 integrase N-terminal mutants.** WT and mutant HIV-1 virion particles were produced in the presence of varying concentrations of the HIV-1 protease inhibitor ritonavir and the cleavage pattern of the Gag and Gag Pol polypeptides were analyzed by immunoblot analysis. WT, methionine substituted (Met) and tryptophan substituted (Trp) integrase mutants were probed with antibodies to integrase (top) or p24 (bottom).

**Figure 8:**
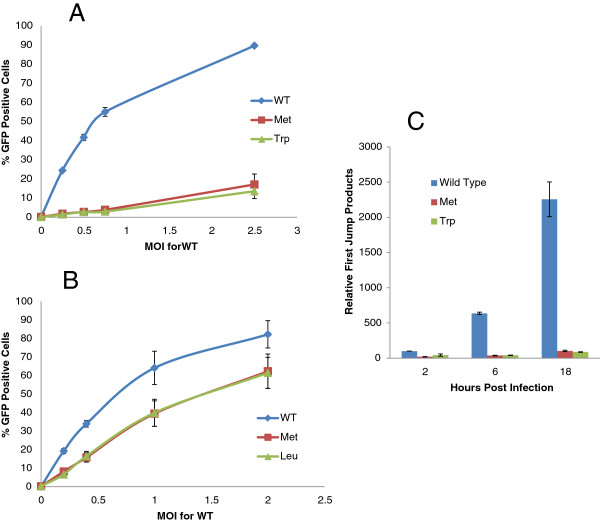
**Effect of N-terminal Integrase mutations on HIV-1 and MLV viral infectivity.** WT or mutant integrase HIV-1 **(A)** or MLV **(B)** viral vectors equilibrated to p24 (HIV-1) or p30 (MLV) amounts were incubated with Jurkat cells and infectivity was measured by flow cytometry 3 days after infection. MOI for WT was measured as indicated in the Methods section and the viral amounts corresponding to equivalent p24 or p30 levels were used for integrase mutants **C**) HIV-1 vectors at an MOI of 1 were incubated with Jurkat cells and viral cDNA first jump products were quantified by qPCR at indicated times. The data are expressed as relative to the control (wild-type) 2 hr time point.

The observed reduction in the amount of integrase and reverse transcriptase suggested a possible defect in the processing of viral Gag Pol polyprotein. To better assess the effect of N-terminal residue integrase mutants of HIV-1 on viral proteolytic processing, we analyzed the intravirion accumulation of Gag, Gag Pol and other proteolytic processing intermediates. We generated WT and integrase mutant virions in the presence of increasing amounts of an HIV-1 protease inhibitor, ritonavir. On probing with a p24 antibody WT and mutant virions showed similar patterns of processing intermediates indicated by the decrease in the level of p24 and p32 followed by an increase in the level of higher molecular weight products such as p55 Gag and p160 Gag Pol as the concentration of ritonavir increased (Figure 7). Gag processing is unaffected by the integrase N-terminal mutations as both WT and mutant virions showed identical patterns of cleavage intermediates (Figure 7, bottom). Virions produced at the highest concentrations of ritonavir (10 μM) contained similar amounts of Gag and Gag Pol suggesting similar levels of packaging of these polyproteins into virions. Probing with an integrase antibody revealed similar processing intermediates p121 or p114 between wild-type and mutant virions (Figure 7, top). The larger proteolytic processing intermediates observed are consistent with previous studies and predictions based on the cleavage sites in the Gag and Gag Pol proteins [27,36]. We have observed an intermediate band between 40 and 50 kDa that appears in the presence of suboptimal ritonavir concentrations. Although this fragment may indicate an alternative cleavage site induced by the mutation at the N terminal residue of Integrase, we have not observed the same fragment in separate virus batches produced in the absence of ritonavir (Additional file 2: Figure S2). Indeed separate experiments with ritonavir have revealed a similar fragment in suboptimal concentrations of ritonavir in both mutants and the WT virus (Additional file 2: Figure S2). Additionally we observe only slight differences in both infectivity and the amount of reverse transcriptase products in different batches of virus preparations (Figure 8A and C). Hence this fragment is a bona fide cleavage intermediate that is not induced by the mutations at the N-terminus of integrase. Collectively these results indicate that protease cleavage site preference is not affected by the mutations at the integrase N-terminal residue. However in the presence of a fully active protease the absolute levels of integrase decreased (compare total signal between WT and mutant virions at 0 μM ritonavir) suggesting that the loss of integrase in the mutants requires HIV-1 protease activity.

### The N-terminal mutants of HIV-1 but not MLV impact infection

Next, we tested the infectivity of these mutants in human cells. As shown in Figure 8A, infectivity was 10–20 folds lower for both HIV-1 integrase mutants compared to wild-type virus. In contrast, MLV integrase mutants exhibited slight decreases in infectivity compared to WT virus in Jurkat cells (Figure 8B). Notably the levels of integrase in the met or trp mutants vary (Figures 6, 7 and Additional file 2: Figure S2) but the levels of infection are equally refractory indicating that the defect is due to a function of the N-terminal amino acid in the RT/uncoating process rather than a defect in proteolytic processing.

Although the main catalytic function of retroviral integrase is to integrate viral double stranded DNA into the host cell chromosome, numerous studies have demonstrated the impact of mutations in the integrase protein on various viral processes including uncoating [37], nuclear import [38,39] and reverse transcription [38,40-44]. In order to determine which step(s) of the viral life cycle is affected by the HIV-1 integrase N-terminal mutants, we measured relative viral DNA accumulation at different time points following infection of the Jurkat cells using quantitative PCR analysis. As shown in Figure 8C, both methionine and tryptophan mutants of HIV-1 integrase showed substantially lower first jump products compared to WT integrase. Approximately 10–20 fold decrease was observed at 6 and 18 hours post infection. These results indicate that the severe inhibition of HIV-1 infection caused by the substitution of the N-terminal residue of viral integrase is the result of a block at the reverse transcription or uncoating step of the viral life cycle.

### N-end rule pathway doesn’t target integrase during retroviral life cycle

**Figure 9:**
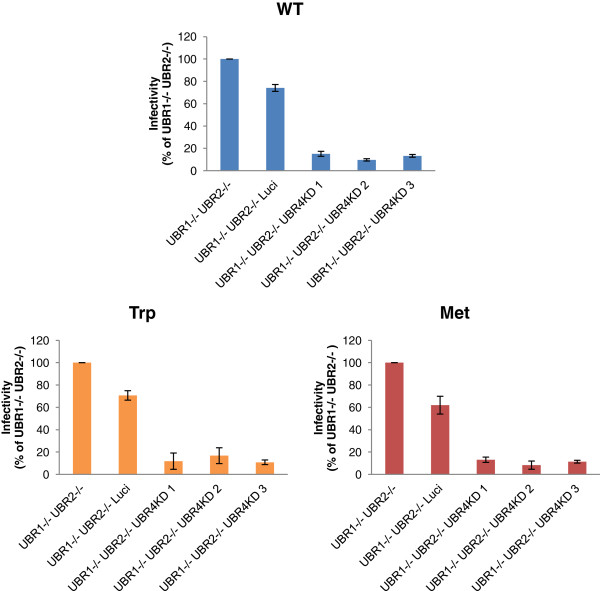
**HIV-1 Integrase N-terminal mutants behave similarly in N-recognin deficient cells.** WT and integrase mutant (Trp and Met) HIV-1 vectors were used to infect N-recognin deficient cells. The infectivity was measured by flow cytometry 3 days post infection and is expressed relative to infection of the UBR1−/− UBR2−/− cell line.

**Figure 10:**
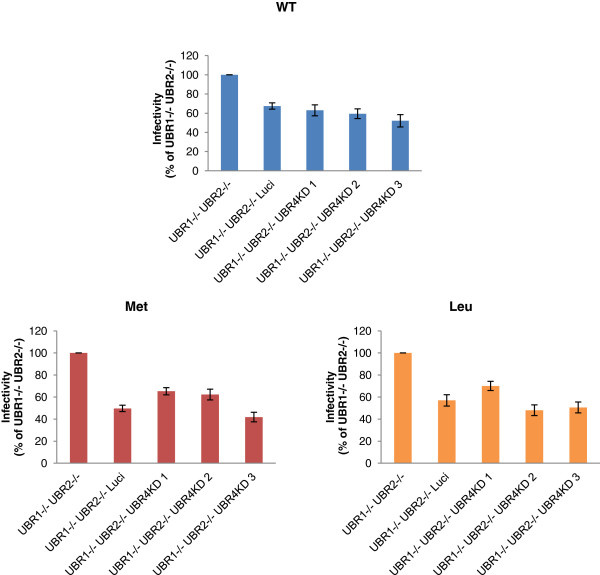
**MLV Integrase N-terminal mutants behave similarly in N-recognin deficient cells.** WT and integrase mutant (Leu and Met) MLV vectors were used to infect N-recognin deficient cells. The infectivity was measured by flow cytometry 3 days post infection and is expressed relative to infection of the UBR1−/− UBR2−/− cell line.

The finding that both stabilizing and destabilizing N-terminal mutants of HIV-1 exhibited similarly decreased infectivity levels supports our hypothesis that integrase is not affected by the N-end rule pathway during viral life cycle. We further tested this hypothesis in experiments using viral mutants and cellular mutants in the N-end rule. If integrase is affected by the N-end rule pathway, the stabilizing N-terminal mutant (methionine) would show similar infectivity on different N-recognin deficient MEFs, while our destabilizing mutant (Tryptophan) would behave like the WT virus. In contrast to these expectations our mutants showed the same pattern in their infectivity of N-recognin deficient MEFs (Figure 9). All HIV-1 mutants showed a 5–6 fold decrease in infectivity when all three ubiquitin ligases are depleted compared to UBR1−/− UBR2−/− cells. In contrast WT MLV and MLV integrase mutants were only slightly decreased and this could be attributed to the overexpression of shRNA since the control luciferase knockdown also resulted in a decrease in infection (Figure 10).

## Discussion

In this study we set out to investigate the role of the N-end rule ubiquitin protease degradation pathway in the retroviral life cycle. This pathway has the potential to act on viral proteins that are generated from polyproteins by protease cleavage. The resultant N-terminal amino acid can vary from the canonical methionine of most proteins and the mature protein can be subject to rapid degradation according to the N-end rule [12]. In looking at HIV-1 by sequence comparison we identified 7 mature proteins that are predicted to have a short half-life according to the N-end rule (Table 1). Comparing 1850 sequences at the Los Alamos HIV Sequence Database that includes all subtypes of HIV-1 and SIV_cpz_ showed an absolute conservation for the N-terminal residue of the mature proteolytically cleaved form of 4 proteins (p1, TFP, RNaseH and Integrase). Figure 1 illustrates this for the integrase sequence. Since N terminal amino acid residue for these 4 proteins is under selective pressure to be conserved, we reasoned the residue at this site might interact with cellular pathways / proteins.

We initially used cell lines that were genetically inactivated for the E3 ligases involved in the N-end rule recognition N-recognins UBR1, UBR2 and UBR4 [18]. From the reported overlap and redundancy in function we utilized cell lines that were multiply inactivated for combinations of the N-recognins. We show that while HIV-1 infection is impacted when all three recognins are inactivated, MLV infection is only marginally affected. This result implied that an N-end rule target protein in the host cell is involved in HIV-1 infection and its stabilization results in a reduction of infection. We also conclude that this protein interacts with HIV-1 but not with MLV and the interaction is not conserved between retroviruses. An alternative hypothesis was that the N-end rule pathway interacts with a protein or proteins from HIV-1 but not MLV. Regardless of the mode of action we determined that the block to infection is at reverse transcription or uncoating.

In order to determine the potential viral target of the N-end rule pathway we decided to examine the role of HIV-1 proteins. We initially focused this study on the integrase molecule because it has previously been shown to be a target for the N-end rule [18,20,21] and it has been a focus for the development of small molecule inhibitors in recent years. Previous data had demonstrated that ectopically expressed integrase with a destabilizing residue was unstable and this instability can be rescued in N-recognin mutants [18]. These studies were extended by using ubiquitin integrase fusions that replaced the N – terminus of integrase with various amino acids and these experiments validated the action of the N-end rule again on ectopically expressed forms of integrase [18]. However these studies did not determine if the integrase was a target for the N-end rule during infection. Hence the question remained whether the N-terminus of integrase was open to interaction with N-recognins in the context of the reverse transcription complex or the pre-integration complex. These are large molecular assemblies in which integrase plays a role. One report did study the N-terminal phenylalanine of integrase in the context of viral infection by mutating the phenylalanine to the stabilizing methionine residue [21]. In this study the authors also mutated the C- terminus of reverse transcriptase to compensate for any defect in proteolytic processing due to the N-terminal substitution. These authors reported that infection was greatly reduced in the methionine (and compensation) mutant and they speculate that this may be due to the stability of integrase by extrapolating the data from the previous reports [18,20]. In this study we included an additional control by mutating the destabilizing WT residue, phenylalanine to another destabilizing and structurally conservative aromatic amino acid tryptophan. We found no absolute defect in proteolytic processing by the single amino acid mutation as reported previously [21]. We found that infection was decreased for both mutations compared to the WT virus (Figure 8A). This result suggests that the severe inhibition of infectivity caused by the amino acid substitution at the N-terminal residue of HIV-1 integrase is not related to the N-end rule pathway. It further confirms that the identity of the N-terminal amino acid of integrase for HIV-1 is vital to its function and explains the tremendous selection pressure that results in this phenylalanine residue being absolutely conserved. This function is unique for HIV-1 since mutating the N-terminal residues of MLV had little impact on infection of MLV.

We further observed that mutating the N-terminal amino acid in integrase resulted in intravirion instability of the integrase molecule (Figure 6). Intravirion instability of the members of the Pol polyprotein has been observed in previous studies [45,46]. We confirm that this instability is in part due to the action of HIV-1 protease since the stability of mutant proteins is restored by inhibiting the protease (Figure 7). This result suggests that HIV-1 protease may degrade the mutant proteins in the virion or that it initiates a cleavage that is acted upon by other proteases packaged virion particles.

Since the N-terminal mutant integrase in the virion still enabled low levels of infectivity, we tested the mutant HIV-1 virions in N-recognin deficient cells. We found that all mutants had a decreased titer on the triply deficient cells compared to the UBR1 −/− UBR2 −/− cells (Figure 9). This reduction was not observed for MLV and mutants at the N-terminus of its integrase (Figure 10). These results support our conclusion that HIV-1 integrase in the context of infection is not a target for the N-end rule pathway.

Our results also point to the importance of the first amino acid phenylalanine of HIV-1 integrase in its function. This residue is often changed using recombinant DNA techniques used to manipulate the integrase coding sequence. Typically this involves adding a methionine start codon to the protein to enable expression of the integrase molecule [47]. Moreover in structural studies residual epitope tags are left as N-terminal extensions after protein purification [48]. Hence future studies should aim to retain the N-terminal phenylalanine especially if integrase is studied in a complex with other proteins. Since *in vitro* assays for integrase function are efficient with recombinant N-terminal methionine bearing molecules, and we have eliminated interaction of the N-terminus with the N-end rule machinery, we speculate that an N-terminal interaction with cellular or viral components involving the phenylalanine is crucial for the early pre-integration phase of HIV-1 infection. Considering the absolute conservation of this residue, this interaction may be a potent target for small molecules to disrupt the viral life cycle.

## Conclusions

We conclude that HIV-1 integrase is not a direct target for the N-end rule pathway mediated degradation during infection. Further the nature of the N-terminal residue of integrase is vital for the infectivity of HIV-1, but not MLV.

## Materials and methods

### Reagents, plasmids and mutagenesis

WT, UBR4 RNAi, UBR1−/−, UBR2−/− and UBR1−/− UBR2−/−, UBR1−/− UBR2−/−UBR4RNAi and UBR1−/− UBR2−/−LuciRNAi cell lines were previously established [18,49,50]. 293T and Jurkat cells were obtained from American Type Culture Collection (ATCC). The following reagents were obtained through the AIDS Research and Reference Reagent Program, Division of AIDS, NIAID, NIH; p24 Monoclonal Antibody (183-H12-5C) from Dr. Bruce Chesebro and Kathy Wehrly, HIV-1 HXB2 Integrase Antiserum (aa 23–34)from Dr. Duane P. Grandgenett, HIV-1 RT Monoclonal Antibody (MAb21) from Dr. Stephen Hughes, Ritonavir. The anti-p30 to MLV was collected from a hybridoma cell line obtained from the ATCC (CRL-1912). Secondary p24 antibody for ELISA was collected from a hybridoma cell line obtained from ATCC (HB-9725). Antibody isolation from the hybridoma cell lines were performed using standard protocols [51]. MLV integrase rabbit polyclonal antibody was a kind gift from Dr. Monica Roth.

Goat-anti-mouse-horseradish peroxidase and goat-anti-rabbit-horseradish peroxidase (HRP) secondary antibodies and West Femto enhanced chemiluminescent (ECL) HRP substrate were obtained from Thermo Scientific (Rockford, IL). Secondary antibody for ELISA; goat-anti-mouse-HRP IgG2A was obtained from Southern Biotech (Birmingham, AL).

Following plasmids were used in this study: CSII-EGFP; an HIV-1 based vector encoding for GFP driven by EF-1a promoter [52]. CSII-DSRed; an HIV-1 based vector encoding for Ds-Red. CSII-CMVGFP; an HIV-1 based vector encoding for GFP driven by CMV promoter. ΔNRF [53]; encodes for gag, pol, rev, tat and vpu of HIV-1. pMDG [54]; encodes for vesicular stomatitis virus glycoprotein. pCLMFG-GFP [55]; and MLV based vector encoding for GFP, pCMVgp [55]; encodes for gag and pol of MLV.

Mutations at the N terminal of HIV-1 and MLV integrase were introduced to ΔNRF and pCMVgp respectively using QuickChange site-directed mutagenesis kit (Stratagene, La Jolla, CA).

### Virus production, infections and culture conditions

HIV-1 and MLV vectors were generated by transient transfection of three plasmids into 293T cells as described previously [54,55]. For HIV-1 vectors 15 μg of CSII EGFP, 10 μg of ΔNRF and 5 μg of pMDG were transfected using the method of Chen and Okoyama [56]. 72 hours after transfection virus was collected and filtered through a 0.45 μM membrane. Following normalization via p24 ELISA, filtered virus was concentrated by ultracentrifugation (100,000 × g, 2 hours at 4°C). Viral pellet was resuspended in Phosphate buffer saline (PBS) and aliquots were stored at −80°C. Viral titers were determined by infecting 1 × 10^5^ Jurkat cells with 10 fold dilutions of the viral preparation. 72 hours after the infection EGFP expression was quantified by flow cytometryon a Becton-Dickinson FACScalibur. Same procedure was followed for production of MLV vectors using following plasmids; 15 μg of pCLMFG-GFP, 10 μg of pCMVgp and 5 μg of pMDG.

MEFs and 293T cells were maintained in Dulbecco’s Modified Eagle Medium (Cellgro) supplemented with 10% Fetal Bovine Serum, FBS (Gemini Bioproducts). Jurkat cells were maintained in Iscove’s Modified Dulbecco’s Medium (ATCC) supplemented with 20% FBS.

### Reverse transcription products qPCR assay

1 × 10^5^ cells were plated into 6 well dishes and infected at an MOI of 1. To control for DNA contamination, the virus was treated with 50 units/mL Benzonase (Sigma) for 1 hour at 37°C before it was added to the cells. Infections were synchronized by incubating the cells 30 minutes at 4°C before and after the addition of the virus. Controls consisted of uninfected cells or cells infected with heat inactivated virus for 36 hours. Cells were harvested and washed with PBS at different time points. Cell lysates were prepared by resuspending the cell pellet in lysis buffer (Tris pH 8.0, 25 mM EDTA pH 8.0, 100 mMNaCl, 1% Triton X-100, and 2 mg/ml proteinase K) and incubating at 55°C overnight. Samples were then incubated at 95°C for 15 minutes to inactivate proteinase K. Lysates were used directly for qPCR analysis. Following primers were used for qPCR [57]: U31 and U32 for first jump products, MHC535 and MHC536 for 2LTR circles: 5' β-actin-ATC ATG TTT GAG ACC TTC AA, 3' β-actin-AGA TGG GCA CAG TGT GGG T, U31 - GGA TCT ACC ACA CAC AAG GC, U32 – GGG TGT AAC AAG CTG GTG TTC, MH535 – AAC TAG GGA ACC CAC TGC TTA AG, MH536 – TCC ACA GAT CAA GGA TAT CTT GTC. QPCR reactions using SYBR green were performed using Eppendorf real plex master cycler ep and BioRad SYBR SuperMix following the manufacturers protocol. Cycling conditions used were 95°C for 2 min, followed by 40 cycles of 95°C 30 s, 58°C 30 s, and 72°C 30 s, and a final extension of 5 minutes at 72°C for all PCR products. Cycle threshold value was used to normalize the DNA amounts to the first time point of the control sample. The melt curve as well as analysis of the PCR products by agarose gel electrophoresis confirmed the presence of one product at the expected size (data not shown). DNA input was controlled by qPCR amplification of a fragment of the β-actin gene.

### Enzyme-linked immunosorbent assay (ELISA)

A 96 well plate (NuncMaxisorp, Fisher Scientific) was coated with a primary p24 antibody (183-H12-5C) in coating buffer (100 mM Sodium Bicarbonate, pH 8.5) and incubated overnight. Wells were then washed 3 times with Phosphate Buffer Saline-Tween (PBS-T) and blocked for 1 hour using 5% milk in PBS-T at room temperature. Following a 3xwash with PBS-T, viral samples were added to the wells at different concentrations in Sodium Chloride-Tris-EDTA (STE) buffer-empigen (0.1 M NaCl, 10 mM Tris–HCl (pH 8.0), 1 mM EDTA (pH 8.0), 0.1% empigen) in triplicates and incubated at 37°C for 1 hour. Wells were then washed 3× with PBS-T and secondary p24 antibody (31-90-25) was added to each well and incubated at room temperature for 1 hour. Following a 3× wash with PBS-T goat-anti-mouse IgG2A-HRP was added to the wells and incubated for 30 minutes at room temperature. Wells were then washed 3× with PBS-T and 3,3′,5,5′-Tetramethylbenzidine, TMB (Sigma) was added to the wells. Following a 10 minute incubation at room temperature, the reaction was stopped with 2N H_2_SO_4_. Absorbance was determined at 450 nm using a microplate reader (BioTEK, SynergyMX).

### Immunoblotting for viral proteins

Filtered virus containing supernatants were concentrated as described above. Following normalization for p24 via ELISA, viral pellets were solubilized in loading buffer (0.25M Tris–HCl, pH 6.8, 15% SDS, 50% glycerol, 25% β-mercaptoethanol, 0.01% bromophenol blue) and proteins were separated by SDS-PAGE on a 12% polyacrylamide gel and subjected to immunoblotting using the antibodies indicated.

### Analysis of intravirionproteolytic processing of HIV-1 polyproteins

293T cells (1 × 10^6^) were transfected using the method of Chen and Okoyama [56] with 15 μg of CSII EGFP, 5 μg of pMDG, and 10 μg of ΔNRF which contains different mutations corresponding to the relevant integrase N-terminal mutant. 8 hours later transfection medium was replaced with culture medium containing different concentrations of the HIV-1 protease inhibitor, ritonavir. 48 hours later virus was collected, filtered and centrifuged at 100,000 × g for 2 hours at 4°C. Purified virions were quantified by p24 ELISA and protein composition of the virions was assessed by immunoblotting as described above.

## Competing interests

The authors declare that they have no competing interests.

## Authors’ contributions

GB carried out the molecular virological studies, carried out the sequence alignment, helped in the design of the study and drafted the manuscript. TT and YTK provided reagents and expertise vital to the study. NVS conceived of the study, participated in its design and coordination and helped to draft the manuscript. All authors read and approved the final manuscript.

## Supplementary Material

Additional file 1: Figure S1 Decrease in infectivity in triply deficient cells is not due to a defect in reporter gene expression. UBR1−/− UBR2−/−, UBR1−/− UBR2−/−UBR4KD and UBR1−/− UBR2−/−Luci RNAi cells were infected with VSVG pseudotyped HIV-1 based vectors encoding either DS-Red or GFP at an MOI of 0.5. The percent of GFP or DS-Red positive cells were determined by fluorescence cytometry 3 days post infection. Click here for file

Additional file 2: Figure S2 Intravirion processing of Gag and Gag-Pol polyproteins in HIV-1 integrase N-terminal mutants. WT and mutant HIV-1 virion particles were produced in the presence of varying concentrations of the HIV-1 protease inhibitor ritonavir and the cleavage pattern of the Gag and GagPol polypeptides were analyzed by immunoblot analysis. WT, methionine substituted (Met) and tryptophan substituted (Trp) integrase mutants were probed with antibodies to integrase. Click here for file
