# Supplementary figures and images for: The N-end rule and retroviral infection: no effect on integrase
Source: Virol J. 2013 Jul 13;10:233. doi: 10.1186/1743-422X-10-233 (PMC3716682; doi:10.1186/1743-422X-10-233)

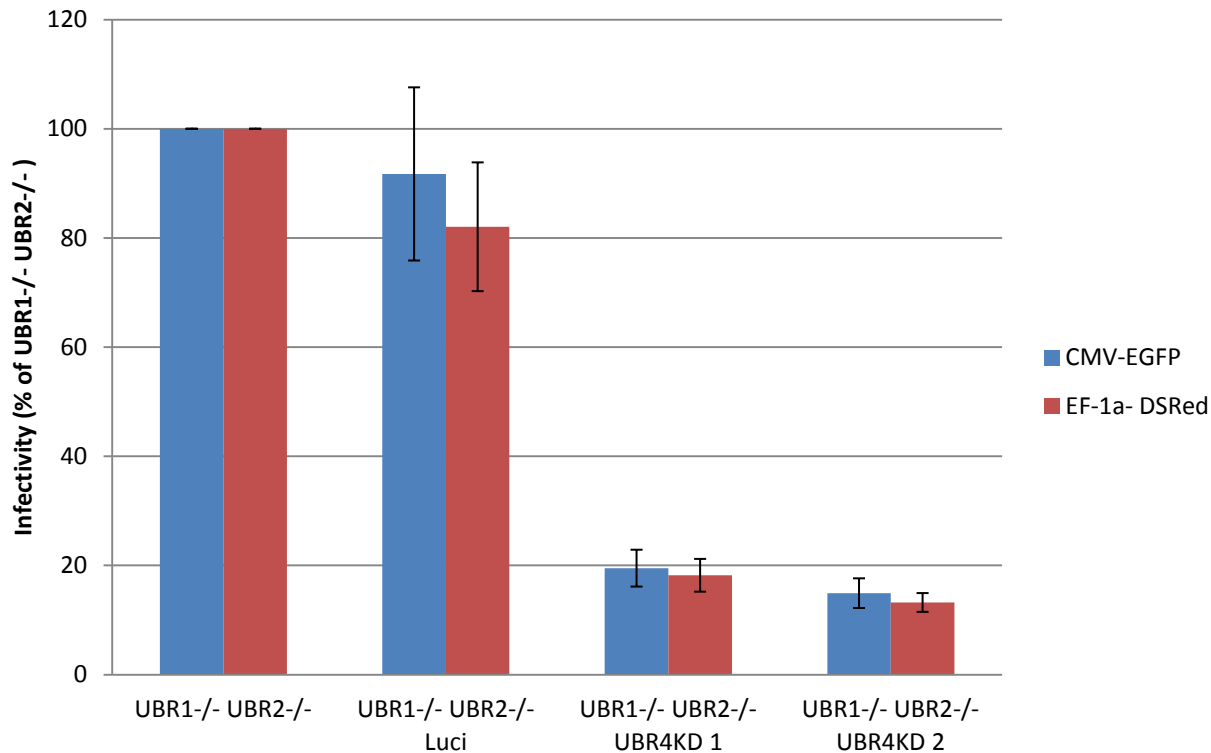

Supplement: Additional file 1: Figure S1 — Decrease in infectivity in triply deficient cells is not due to a defect in reporter gene expression. UBR1−/− UBR2−/−, UBR1−/− UBR2−/−UBR4KD and UBR1−/− UBR2−/−Luci RNAi cells were infected with VSVG pseudotyped HIV-1 based vectors encoding either DS-Red or GFP at an MOI of 0.5. The percent of GFP or DS-Red positive cells were determined by fluorescence cytometry 3 days post infection. [file 1743-422X-10-233-S1.pdf]

kDa

Trp

Met

WT

0  $\mu$ M 0.5  $\mu$ M 1  $\mu$ M 10  $\mu$ M 0  $\mu$ M 0.5  $\mu$ M 1  $\mu$ M 10  $\mu$ M 0  $\mu$ M 0.5  $\mu$ M 1  $\mu$ M 10  $\mu$ M

150  
100  
80  
60  
50  
40  
30  
25

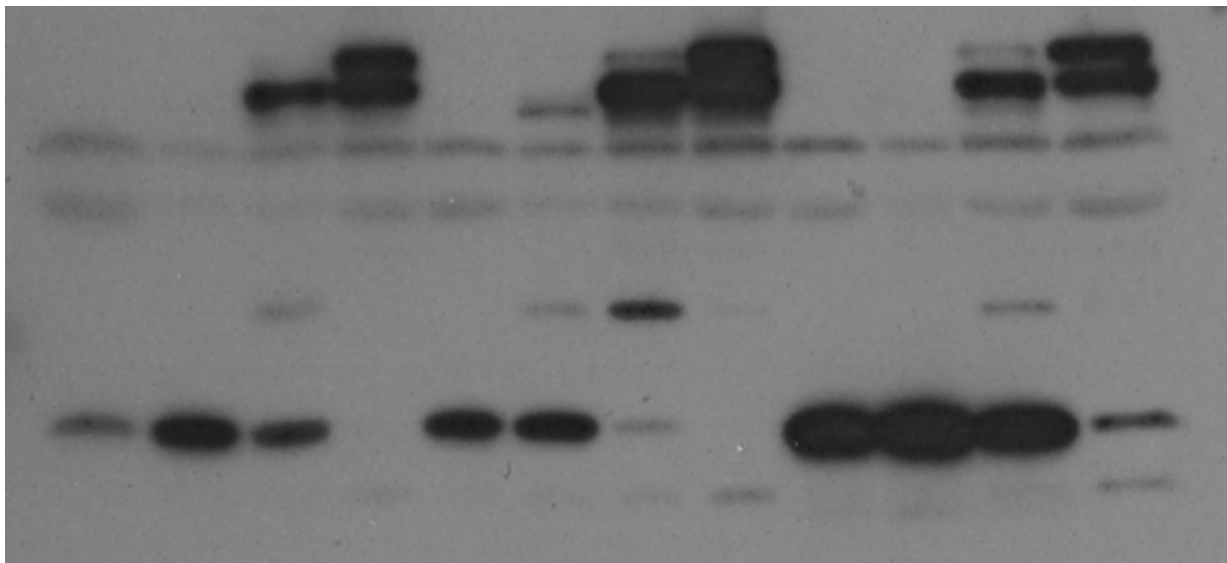

Supplement: Additional file 2: Figure S2 — Intravirion processing of Gag and Gag-Pol polyproteins in HIV-1 integrase N-terminal mutants. WT and mutant HIV-1 virion particles were produced in the presence of varying concentrations of the HIV-1 protease inhibitor ritonavir and the cleavage pattern of the Gag and GagPol polypeptides were analyzed by immunoblot analysis. WT, methionine substituted (Met) and tryptophan substituted (Trp) integrase mutants were probed with antibodies to integrase. [file 1743-422X-10-233-S2.pdf]
